# Lupins as Plants Poisonous to Stock

**Published:** 1899-12

**Authors:** E. W. Wilcox

**Affiliations:** Department of Agriculture, Washington, D. C.


### LUPINS AS PLANTS POISONOUS TO STOCK.

By E. W. Wilcox, Ph.D.,
DEPARTMENT OF AGRICULTURE, WASHINGTON, D. C.

The study of poisonous plants is a subject which is accompanied with certain peculiar difficulties. These difficulties are not insurmountable, but they render progress slow in this line of work. When the farmer finds some of his stock dead he attempts to discover the cause. If there seems to be no other reasonably assignable cause some plant is usually suspected. Specimens of the plant, or perhaps the contents of the stomach of a dead animal, are then sent to a chemist or veterinarian. The account of the conditions under which the animals were found just previous to their death is usually meagre and unsatisfactory. By the time the investigator arrives on the scene the trouble is past. The owner of the animals shows him some decomposing carcasses and certain plants growing near at hand which are supposed to have poisoned the animals. From these data the investigator is supposed to be able to diagnose the whole matter and suggest suitable preventions or remedies. I have known of the contents of sheep stomachs being sent for analysis ten days after death. I was once called to investigate a supposed poisoning of horses, and found three carcasses of horses which had been dead over two weeks. The horses had ranged over a tract of about 200 acres, yet the farmer seemed surprised that I could not determine the cause of the death of his horses.

My attention was first drawn to our native lupins as possible poisonous plants, in Montana, in the fall of 1896. Lupins are used for hay in Montana to a considerable extent. The more common species are *Lupinus leucophyllus* and *L. plattensis.* These plants, particularly the former species, are widely distributed in the State. Large areas of them are cut for hay, and I have seen many stacks of nearly clean lupin hay. In this condition it is readily eaten by horses, cattle, and sheep. Probably not less than 5000 tons of lupin hay are fed annually in Montana.

Although these plants are thus used extensively for fodder, it has long been believed that they are poisonous under certain circumstances. There seems to be no good reason for doubting this belief. I have myself witnessed or have gotten a definite description of a number of disastrous cases of sheep-poisoning where it appeared impossible to avoid the conclusion that lupin was the cause of the trouble.

It may be well briefly to relate the circumstances of some of these cases.

In August, 1896, a band of sheep while being driven from one range to another was allowed to stop and feed in a field of lupin. Within two hours' time a number of the sheep showed symptoms of serious illness, and of the 200 sheep in the band 100 had died before the following morning. The lupin pods were fully formed at the time, but the seeds were not quite ripe. No bloating was manifested until after death. The symptoms were such as usually appear in lupin-poisoning as described by European authors, and will be mentioned shortly.

One afternoon in the winter of 1897 a band of 150 bucks which were kept in a covered corral was given a liberal quantity of lupin hay. Previous to this time the sheep had received cultivated hay. The lupin was given to the sheep at 3 o'clock P.M. About three hours later the owner became aware of an unusual disturbance among the sheep. Upon arriving at the corral he found the majority of the sheep in a frenzied condition, and during the night about 90 of them died. Other hay was bought at once and no further trouble was experienced.

During the same winter three two-year-old colts which had been receiving cultivated hay as fodder were fed lupin hay for two days. All three died on the second day.

A somewhat similar case came under my observation, of a horse which had eaten lupin hay about two hours before I arrived. The owner of the horse had bought the hay earlier in the day, and, as he was not familiar with lupin, he did not recognize it in the form of hay. The horse became violently ill, but was given no more lupin, and recovered after two or three days.

During the month of October, 1898, unusually serious losses of sheep occurred in Montana. About 2000 sheep died in different parts of the State. Out of one band of 2500 about 1150 died. These last-mentioned cases occurred just after a snow-storm, when the short grass of the ranges was covered and the sheep were forced to eat the lupin, which stood up temptingly above the snow. We examined the stomach contents of ten of these sheep and found that lupin was almost the only food material. Stems, leaves, pods, and seeds could be easily identified.

Beside the observations which we ourselves have made we know of no work in this country to determine the poisonous or non-poisonous character of our native lupins with the exception of Dr. S. B. Nelson's single experiment. He fed one sheep eight and a quarter pounds of lupin within three days without any poisonous effects being manifested. The experiment, however, was conducted in the month of May, and it should be remembered that the lupins are green at that season and could not possibly be in fruit. It was already well known to sheep raisers that lupin is freely eaten in its younger stages by sheep without bad consequences, and that lupin hay when cut before the fruiting period is often quite harmless. The chemical composition of all plants which have been carefully studied undergoes numerous and important changes during the various stages of growth. Many characteristic drugs and poisons are found only in certain parts of the plant, as, for example, in the roots or seeds. As is well known to pharmacists, most medicinal plants yield the desired drugs only when collected at the proper season. From such obvious facts as these alone it should be apparent that conclusions drawn from experiments with plants in only one stage of their growth must be limited to their application.

It has long been known that the European species of lupins contain poisonous principles. Dioscorides, Cato, Pliny, Columella, and Theophrastus give good accounts in their writings of the use of lupins as food for man and animals, and describe the methods by which the poisonous principles are removed from the seeds so as to render them safe. Since that time a rather voluminous literature of the subject has been developed in Europe. This literature covers the medicinal use of lupins (Pliny enumerated thirty-five different medicinal uses of these plants), their value as green manure, their use as forage plants, and as food for man. A great amount of work has been done on the chemistry of lupins, and numerous methods have been devised and patented for the extraction of the poisonous alkaloids, especially from the seeds, in order to render them safe for food.

The European species of lupins have caused extensive poisoning of stock, particularly of sheep, and there has been much discussion as to just which constituents of the lupins produce the poisonous effects. A number of alkaloids have been isolated and carefully studied from chemical and pharmacological stand-points.

The symptoms of sheep-poisoning by lupins are well known, and the disease is ordinarily called lupinosis in Europe. It may be of either an acute or lingering form, and the sheep live from a few hours to about three days. The symptoms, as I have observed them in poisoning from our native species of lupins, are practically identical with those described by European writers. There is usually great cerebral congestion, accompanied first with mental excitement, during which the sheep rush frantically about, butting one another and other objects. This stage is followed by locomotor ataxia, spasms, convulsions, and collapse. In the lingering form of the poisoning I have observed a marked weakening of the pulse and slowness of respiratory movements. In one case in a horse I noticed an increase in the amount of urine and in the frequency of urination.

There are a number of native species of lupins which grow abundantly at all altitudes throughout the Rocky Mountain States. They are eaten by stock to a greater or less extent at all stages of growth. The California Agricultural Experiment Station has been experimenting for the past seven years with species of European and native lupins for the purpose of determining their value as green manures. Only two native species gave promise of value for agricultural purposes, *Lupinus affinis* and *L. micranthus.*

Lupins may be considered quite important native forage plants. They are actually used to a considerable extent for hay, and seem to furnish a safe and nutritious fodder if cut before the seeds are ripe. They have, however, caused large losses of stock when used in a ripe condition. It would seem unwise to recommend the cultivation of lupins for forage, and the fact that the poisonous alkaloids must be extracted from the seeds before they can be used as food for man would probably make their cultivation for this purpose unprofitable in this country. For green manures some of the lupins have been found well adapted, but no more so than many other leguminous plants. The seeds of lupins are always poisonous and the foliage appears to be poisonous under certain circumstances. In localities where losses of stock occur from lupin-poisoning, it seems proper to recommend that stock-raisers should put less dependence in dangerous lupin hay and cultivate standard forage crops for winter-feeding.

So far as is known to the writer there has been no chemical work done upon our native lupins. They are plants of considerable agricultural importance in the Rocky Mountain States, both as native forage and as the cause of great losses of stock. The present paper is prepared for the purpose of calling attention to the importance of the subject, and of giving a list of the related literature, which will, it is hoped, be of help to chemists and veterinarians who may take up this work.

The bibliography which follows includes all the books and articles which I have consulted in the study of lupins. It will be seen that the titles cover a considerable variety of topics, including botanical, agricultural, chemical, physiological, and pharmacological studies of lupins. I make no sort of claim to its being complete, but shall be grateful for the receipt of references not included in my list.

### Literature Relating to Lupins.

Agardh, J. G. Synopsis generis Lupini. Lund, 1835.

Ahrendt, A. Verfahren zur Entbitterung von Lupinen und Herstellung von Lupinen kuchen. Ber. Deut. Chem. Gesell., Referat 24 (1891), p. 174.

Allen, E. W. Leguminous Plants for Green Manuring and for Feeding. U.S.D.A., Farmers' Bul., 16, 1894.

Arnold, C. Isolierung des in gewissen Lupinen enthaltenen giftigen Stoffes. Ber. Deut. Chem. Gesell., 16 (1883), pp. 461, 462.

Barral et Sagnier. Dictionnaire d'agriculture. Paris, 1889, vol. iii. pp. 581, 582.

Baumert, G. Ueber das flüssige Alkaloid aus Lupinus luteus. Liebig's Ann. Chem., 224 (1884), pp. 321–330.

——— Ueber das Verhalten des Lupinens zu wasserentziehenden Agentien. Liebig's Ann. Chem., 214 (1882), pp. 361–376.

——— Ueber das Verhalten des Lupinidins zu Aethyljodid. Liebigs's Ann. Chem., 227 (1885), pp. 207–220.

——— Einwirkung von Acetylchlorid und Essigsäure-anhydrid auf Lupinin. Liebig's Ann. Chem., 224 (1884), pp. 313–321.

——— Das Lupinidin aus Lupinus luteus. Liebig's Ann. Chem., 225 (1884), pp. 365–384.

——— Das Lupinin. Landw. Vers. Stat., 27 (1882), pp. 15–64.

——— Weitere Untersuchungen über den flüssigen Theil der Alkaloide aus Lupinus luteus: Lupinidin. Landw. Vers. Stat., 31 (1885), pp. 139–153.

——— Einwirkung von Natrium auf Lupinen. Ber. Deut. Chem. Gesell., 15 (1882), pp. 631-633.

——— Anhydrolupinin. Ber. Deut. Chem. Gesell., 15 (1882), pp. 634–636.

——— Verarbeitung der Lupininrückstände auf Salzsäures Lupinin. Ber. Deut. Chem. Gesell., 15 (1882), pp. 1951, 1952.

——— Zur Kenntniss der Lupinenalkaloide. Ber. Deut. Chem. Gesell., 14 (1881), pp. 1150–1153, 1321–1323, 1880–1884.

——— Das Löhnert'sche Lupinenentbitterungsverfahren. Ber. Physiol. Lab. Landw. Inst. Univ. Halle, Dresden, 1895, No. 12, pp. 48–51.

——— Untersuchungen über den flüssigen Theil der Alkaloide aus Lupinus luteus. Landw. Vers. Stat., 30 (1884), pp. 295–330.

——— Zur Quantitativen Bestimmung des Alkaloidgehaltes der Lupinen. Chem. Ztg., 8 (1894), pp. 137 and 195.

Beaurredon, Abbé. Voyage agricole chez les Anciens. Paris, 1898, pp. 94–96.

Bellini, R. Danger de l'emploi des graines du lupin comme vermifuge. Journ. Pharm. et Chim., 4 ser., 27 (1878), p. 465 (abstract).

——— Action toxique du lupin. Journ. Pharm. et Chim, 5 ser., 6 (1882), pp. 203–204 (abstract).

——— Dell' Avvelenamento prodotto nell'uomo e nei bruti della decozione dei semi del lupino. Lo Sperimentale, 35 (1875), pp. 260–271.

Bente, F. Verfahren zur Entbitterung der Lupinen. Biedermann's Centbl., 14 (1885), pp. 91, 92.

Berthelot et André. Nouvelles recherches sur la marche général de la végétation. Ann. Chim. et Phys., 7 ser., 9 (1896), pp. 5–119.

Beyer, A. Ueber einige Bestandtheile des gelben Lupinen-Samens. Landw. Vers. Stat., 14 (1871), pp. 161–176.

——— Ueber den Bitterstoff der gelben Lupine. Landw. Vers. Stat., 10 (1868), pp. 518–520.

——— Ueber die Keimung der gelben Lupine. Landw. vers. Stat., 9 (1867), pp. 168–178.

Birnbaum, E. Pflanzenbau. Berlin, Paul Parey, 1898, pp. 65-70, figs. 2.

Bitto, B. v. Ueber die Bestimmung des Lecithingehaltes der Pflanzenbestandtheile. Ztschr. Physiol. Chem., 19 (1894), pp. 488–498.

Blomeyer, A. Die Kultur der landwirthschaftlichen Nutzpflanzen. Leipzig, 1891, vol. i.

Bordeau, L. Conquête du monde Végétal. Paris, 1893, p. 73.

Buddenbrock, B. von. Ueber Lupinose. Deut. Landw. Presse, 8 (1881), p. 104.

Campani, G., et Grimaldi, S. La vanillina nei semi del Lupinus albus. L'Orosi, 11 (1888), pp. 43–45; Gaz. Chim. Ital., 17 (1887), pp. 545–547.

——— Contribuzione alle conoscenze chimische sui semi del lupino bianco. L'Orosi, 11 (1888), pp. 263–269; Gaz. Chim. Ital., 18 (1888), pp. 436–442.

——— Sulla lupinidina del lupino bianco (L. albus). L'Orosi, 14 (1891), pp. 19–24; Gaz. Chim. Ital., 21 (1891), pp. 432-437.

Campani, G. Sul principio venefico dei semi del Lupino commune (L. albus). L'Orosi, 1881, pp. 2–15; Gaz. Chim. Ital., 11 (1881), pp. 237–240.

Carter, J. M. G. A Synopsis of the Medical Botany of the United States. St. Louis, 1888, p. 176.

Cato. De re rustica. Leipzig, J. M. Gerner, 1735.

Chesnut, V. K. Preliminary Catalogue of Plants Poisonous to Stock. U. S. Dept. Agr., Bureau of Animal Industry Rpt., 1898, pp. 387–420.

Columella. De re rustica. London, 1745, pp. 71, 72.

Cornevin, C. Des Plantes Vénéneuses et des Empoisonnement qu'elles déterminent. Paris, 1893, p. 524.

Crescenzi, Pietro de. D'Agricoltura. Venice, 1542, book 3, chap. 14.

Dammann, Dr. Ueber Schafheerden-Erkrankungen durch Lupinen. Deut. Ztschr. Thiermed., 3 (1877), pp. 353–367.

Davis, L. S. Ueber die Alkaloide der Samen von Lupinus albus und L. angustifolius. Inaugural Dissertation. Marburg, 1896.

Davy, J. B. Lupins for Green-manuring. California Sta. Bul., 124, p. 31.

De Candolle, A. Origin of Cultivated Plants. New York, 1892.

Deloncle et Dubreuil. Dictionnaire populaire d'agriculture pratique. Paris, fascicle 7, pp. 1078, 1079.

Denaiffe, C. and H. Manuel Pratique de Culture Fourragère. Paris, 1896.

Dickson, A. The Husbandry of the Ancients. Edinburgh, 1788, vol. ii. p. 181.

Dioscorides. De materia medica. Basel, 1529, pp. 119, 120.

Donnabella, R. Sul azione venefica dei lupini usati come mezzo vermicida. Il Morgagni, 19 (1887), pp. 453–456.

Dragendorff, G. Die Heilpflanzen der verschiedenen Völker und Zeiten. Stuttgart, F. Enke, 1898, pp. 884.

Eichhorn. Ueber das Lupinin. Landw. Vers. Stat., 9 (1867), pp. 272–276.

Frank, B. Ueber die Pilzsymbiose der Leguminosen. Landw. Jahrb., 1890, pp. 523–640, pls. 3.

Fruwirth, C. Anbau der Hülsenfrüchte. Berlin, Paul Parey, 1898, pp. 122–143, figs. 9.

Gabriel, S. Zur Kenntniss der Rohfaserbestimmung. Ztschr. Physiol. Chem., 16 (1892), pp. 370–386.

Hagen, M. Ueber das Lupanin, ein Alkaloid aus dem Samen der blauen Lupine (L. angustifolius). Liebig's Ann. Chem., 230 (1835), pp. 367–384.

Heiden, E. Aschen-analysen von Lupinen. Landw. Vers. Stat., 8 (1866), pp. 455–458.

Heidepriem, F. Fütterungsversuche mit Schafen. Landw. Vers. Stat., 16 (1873), pp. 1–401.

Heldreich, T. Die Nutzpflanzen Griechenlands. Athens, 1892.

Henzé, G. Les plantes Fourragères. Paris, 3 ed.

Hildebrand, A. Handbuch des landwirthschaftlichen Pflanzenbaues. Berlin, Paul Parey, 1889, p. 484, figs. 233.

Hilgard, E. W. Supply of Soil Nitrogen. California Sta. Rpt., 1894–95, pp. 32–35.

——— Crops for Green-manuring. California Sta. Rpt., 1894–95, pp. 118–133.

Hiller, E. Ueber den Alkaloidgehalt verschiedener Lupinenarten und Varietäten. Landw. Vers. Stat., 31 (1885), pp. 336–341.

Hlubek, F. X. Die Landwirthschaftslehre. Vienna, 1 (1853).

Jacobson, H. Ueber einige Pflanzenfette. Ztschr. Physiol. Chem., 13 (1889), pp. 32–65.

Jungman, C. Verfahren zur Bereitung eines Kaffeesurrogates aus Lupinen. Ber. Deut. Chem. Gesell., Referat 26 (1893), p. 122.

Kellner, O. Zur Entbitterung der Lupinen. Deut. Landw. Presse, 23 (1896), p. 271.

——— Versuche über die Entbitterung und Verdaulichkeit der Lupinenkörner. Landw. Jahrb., 9 (1880), pp. 977–998.

Kette, W. Die Lupine als Feldfrucht. Berlin, 1891, p. 124.

Kühn, J. Zur Erhaltung des Culturwerthes der Lupine. Deut. Landw. Presse, 8 (1881), pp. 162, 163.

——— Das Einsäuren (Einmachen) der Futtermittel. Berlin, 1894, pp. 1–74.

——— Die Smarotzerpilze der Lupinenflanze und die Bekampfung der Lupinenkrankheit der Schafe. Centbl. med. Wiss., 19 (1881), p. 498.

Langethal, C. E. Klee und Wickpflanzen. Berlin, 1874, 5 ed.

Lawes, J. B., and Gilbert, J. H. The Sources of Nitrogen of our Leguminous Crops. Journ. Roy. Agr. Soc. England, 2 (1892), 3d ser., No. 4, pp. 657–702.

Lewin, L. Lehrbuch der Toxikologie. Wien u. Leipzig, 1898, p. 509.

Liebscher, G. Beitrag zur Klarlegung der Frage nach den Ursachen der Lupinenkrankheit der Schafe. Centbl. med. Wiss., 19 (1881), pp. 497, 498.

Likiernik. A. Lupeol. Ber. Deut. Chem. Gesell., 24 (1891), pp. 183, 2709.

Löhnert, W. Zur Entbitterung der Lupinen. Deut. Landw. Presse, 23 (1896), p. 311.

McCarthy, G. Some Unsuccessful Legumes. Country Gentleman, 64 (1899), No. 2438, p. 824

Maisch, J. M. Materia Medica of the Mexican Pharmacopœia. Amer. Journ. Pharm., 57 (1885), p. 533.

Merlis, M. Zusammensetzung der Samen und etiolierten Keimpflanzen von Lupinus angustifolius. Landw. Vers. Stat., 48 (1897), p. 418.

Müller, F. von. Select Extra-tropical Plants. Sydney, Thomas Richards, 1881.

Münsberg, B. Verfahren zur Entbitterung von Lupinen und zur gleichzeitigen Entfernung der in ihnen enthaltenen Giftstoffe. Ber. Deut. Chem. Gesell., Referat 25 (1892), p. 707.

Naudin, C., and Müller, F. von. Manuel de l'acclimateur ou choix des plantes reccommandées pour l'agriculture l'industrie et la médecine. Paris, 1887.

Nelson, S. B. Feeding Wild Plants to Sheep. U. S. Dept. Agr., Bureau of Animal Industry Report, 1898, pp. 421–425.

Nobbe, F., Schmidt, E., Hiltner, L., and Hotter, E. Versuche über die Stickstoffassimilation der Leguminosen. Landw. Vers. Stat., 39 (1891), pp. 327–359.

Pabst, H. W. Lehrbuch der Landwirthschaft. Vienna, 1 (1865), pp. 379–382.

Palladius. De re rustica.

Pasqualini, A. Del modo piu conveniente per toglieri l'amaro ai lupini polverizzarli con la minor perdita possibile di materie albuminiche ed utilzzarli come foraggio. Staz. Sper. Agr. Ital., 29 (1896), pp. 917–927.

Piana, G. Sul lupino bianco. Bul. Sci. Med., 7 (1881), pp. 180–183.

Pickering, C. Chronological History of Plants. Boston, Little, Brown & Co., 1879.

Pinckert, F. A. Die Lupine; Anleitung zu deren Cultur und Benützung als bodenbereichernde Samen-, Futter-, und Gründüngungspflanze. Berlin, Schotte & Co., 1860, p. 91.

Pliny. Natural History, vol. iv.

Pott, E. Die Landwirthschaftliche Futtermittel. Berlin, Paul Parey, 1889, p. 730.

Richard, A. Dictionnaire raisonné d'agriculture. Paris, 1855, vol. ii. pp. 89, 90.

Ridolfi, C. Lezioni orali di Agraria. Florence, 2 (1862). pp. 38–41.

Ritthausen, H. Ueber das Pflanzen-Casein oder Legumin. Jour. Prakt. Chem., 103 (1868), pp. 65–85.

——— Ueber die Säuren der Samen der galben Lupinen (L. luteus). Jour. Prakt. Chem., 110 (1870), pp. 339–347.

——— Ueber die Einwirkung von Salzlösungen auf Conglutin und Legumin. Jour. Prakt. Chem., 132 (1881), pp. 221–225.

——— Ueber das Verhalten des Conglutins aus Lupinensamen zu Salzlösungen. Jour. Prakt. Chem., 134 (1882), pp. 422–440.

——— Zur Darstellung der Alkaloide der gelben Lupinen (L. luteus). Chem. Ztg., 21 (1897), No. 2, p. 718.

——— Ueber Galactit aus dem Samen der gelben Lupine. Ber. Deut. Chem. Gesell., 29 (1896), pp. 896–899.

Roloff. Acute Gelbsucht der Schafe. Centbl. med.Wiss., 19 (1881), pp. 593, 594.

Rozier, Abbé. Cours complet d'Agriculture. Paris, 1785, vol. vi. pp. 330–335.

Schmidt, E. Ueber die Alkaloide der Lupinensamen. Pharm. Centr., 37 (1896), pp. 538, 539.

Schulze, B. Zur Entbitterung der Lupinen. Deut. Landw. Presse, 23 (1896), p. 377.

Schulze, E., and Bosshard, E. Zur Kenntniss des Vorkommens von Allantoin Asparagin Hypoxanthirom. und Guanin in den Pflanzen. Ztschr. Physiol, Chem., 9 (1885) pp. 420–444.

Schulze, E. Bilden sich Cholesterine in Keimpflanxen welche bei Lichtabschluss sich entwickeln? Ztschr. Physiol. Chem., 14 (1890), pp. 491–521.

Schulze, E., and Steiger, E. Ueber das Lecithingehalt der Pflanzensamen. Ztschr. Physiol. Chem., 13 (1899), pp. 365–384.

Schulze, E., Steiger, E., and Maxwell, W. Untersuchungen über die chemische Zusammensetzung einiger Leguminosensamen. Landw. Vers. Stat., 39 (1891), pp. 269–326.

Schulze, E. Berichtigung. Jour. Prakt. Chem., 28 (1883), p. 63.

Schulze, E., and Barbieri, J. Ueber Phenylamidopropionsäure, Amidovaleriansäure und einige andere stickstoffhaltige Bestandtheile der Keimlinge von Lupinus luteus. Jour. Prakt. Chem., 27 (1883), pp. 337–362.

——— Ueber ein neues Glucosid (Bestandtheil von Lupinus luteus). Ber. Deut. Chem. Gesell., 11 (1878), pp. 2200–2203.

Schulze, E., and Steiger, E. Ueber das Arginin. Ztschr. Physiol. Chem., 11 (1887), pp. 43–65.

Schulze, E. Untersuchungen über die zur Klasse der stickstoffhaltigen organischen Basen gehörigen Bestandtheile einiger landwirthschaftlich benutzten Samen, Oelkuchen und Wurzelknollen sowie einiger Keimpfianzen. Landw. Vers. Stat., 46 (1895), pp. 23–77.

——— Zur Kenntniss des B-Galactans. Ber. Deut. Chem. Gesell., 25 (1892), pp. 2213–2218.

Schulze, E., and Steiger, E. Ueber Paragalactin. Ber. Deut. Chem. Gesell., 20 (1887), pp. 290–294.

Schulze, E. Ueber das Verhalten der Lupinenkeimlinge gégen distillirtes Wasser. Landw. Jahrb., 1891, p. 236.

Schulze, E., and Steiger, E. Ueber einen neuen stickstoffhaltigen Bestandtheil der Keimlinge von L. luteus. Ber. Deut. Chem. Gesell., 19 (1886), pp. 1177–1180.

Schulze, E. Ueber die Zellwandbestandtheile der Cotyledonen von L. luteus und L.

angustifolius, und über ihr Verhalten während des Keimungsvorganges. Ztschr. Physiol. Chem., 21 (1896), pp. 392–411.

Schulze, E., and Barbieri, J. Zur Kenntniss der Cholesterine. Jour. Prakt. Chem., 25 (1882), pp. 159–180.

——— Ueber das Vorkommen von Phenylamidopropionsäure unter den Zersetzungsprodukten der Eiweisstoffe. Ber. Deut. Chem, Gesell., 14 (1881), pp. 1785–1791.

Schulze, E., and Steiger, E. Untersuchungen über die stickstoffreien Reservestoffe der Samen von L. luteus, und über die Umwandlungen derselben während des Keimprocesses. Landw. Vers. Stat., 36 (1889), pp. 391–476.

Schulze, E. Zur Kenntniss der in den Leguminosensamen enthaltenen Kohlendydrate. Landw. Vers. Stat., 41 (1892), pp. 207–229.

Schulze, E., and Likiernik, A. Ueber das Lecithin der Pflanzensamen. Ztschr. Physiol. Chem., 15 (1891), pp. 405–414.

Schulze, E., Steiger, E., and Maxwell, W. Zur Chemie der Pflanzenzellmembranen. Ztschr. Physiol. Chem., 14 (1890), pp. 227–273.

Schulze, E. Ueber das Vorkommen von Cholin in Keimpflanzen. Ztschr. Physiol. Chem., 11 (1887), pp. 365–372.

——— Untersuchungen über die Amidosäuren welche bei der Zersetzung der Eiweisstoffe durch Salzsäure und durch Barytwasser entstehen. Ztschr. Physiol. Chem., 9 (1885), pp. 63–126.

Schnuck, E., and Marchlewski, L. Studien über einige natürliche Zuckerarten. Liebig's Ann. Chem., 278 (1894), pp. 349–359.

Siebert, C. Ueber das Lupanin, das Alkaloid der blauen Lupine. Arch. Pharm., 229 (1891), pp. 531–546.

Siewert, M. Ueber die Alkaloide der Lupinus-arten. Landw. Vers. Stat., 12 (1869), pp. 306–316, 321–344.

Simpson, R. Anleitung zur vollständigen Entbitterung der blauen Lupine. Mohrungen, 1885, p. 15.

Smith, John. Domestic Botany. London: Macmillan & Co., 1883.

Soldaini, A. Sopra i prodotti di scompósizione del composto bromurato dell 'alcaloide deliquescente del Lupinus albus. L'Orosi, 18 (1895), pp. 37-48.

——— Sopra alcuni metodi di estrazione degli alcaloide dei semi di Lupinus albus. L'Orosi, 18 (1895), pp. 73–86

——— Sugli alcaloide del Lupinus albus. L'Orosi, 20 (1897), pp. 109–113.

——— Sopra gli alcaloide del Lupinus albus. L'Orosi, 15 (1892), pp. 115–118.

——— Sopra gli alcaloide dei semi di Lupinus albus. L'Orosi, 15 (1892), pp. 325–348.

——— Ricerche sulla costituzione dell alcaloide deliquescente del Lupinus albus. L'Orosi, 16 (1893), pp. 109–126.

——— Nuovi composti degli alcaloidi del Lupinus albus. L'Orosi, 17 (1894), pp. 253–262.

——— Sopra gli alcaloide dei semi di Lupinus albus. Gaz. Chim. Ital., 23 (1893), pt. 1, pp. 143–168.

Soltsien, P. Verfahren zur Entbitterung von Lupinen und anderen Fruchten. Ber. Deut. Chem. Gesell., Referat 19 (1886), pp. 805, 806.

Steiger, E. Ueber B-Galactan ein dextrinartiges Kohlehydrate aus den Samen von L. luteus. Ztschr. Physiol. Chem., 11 (1887), pp. 373–387.

——— Ueber das dextrinartiges Kohlehydrat der Samen von Lupinus luteus. Ber. Deut. Chem. Gesell., 19 (1886), pp. 827–830.

Steiner, L. Ueber Entbitterung und Entgiftung der Lupinenkörner. Inaugural dissertation. Halle, 1894, p. 32.

Täuber, E. Ueber den Alkaloidgehalt verschiedener Lupinenarten und Varietäten. Landw. Vers. Stat., 29 (1883), pp. 251–257.

Theophrastus. Historia plantarum and De causis plantarum.

Tommasi, T. Nuove ricerche sull'azione tossica del Lupino. L'Imparziale, 22 (1882), pp. 759–761.

Trabut, L. Précis de Botanique médicale. Paris, 1898, 2 ed., p. 739, figs. 954.

Varro. De re rustica, in Scriptores Rei rusticæ Aeteres Latini. Leipzig, J. M. Gesner, 1735.

Vesque, J. Traité Botanique agricole et industrielle. Paris, 1885.

Vogl, A. E. Nahrungs- und Genussmittel. Berlin, 1899, p. 575.

Wächter, G. Ueber die Entbitterung der Lupinen. Inaug. Diss. Leipzig, 1892.

Weiske, H. Ueber das Entbittern der Lupinenkörner. Jour. Landw., 36 (1888), pp. 445–454.

Werner, H. Handbuch des Futterbaues, Berlin, Paul Parey, 1889, 2 ed.

Wilcox, E. V. Larkspur Poisoning of Sheep. Montana Sta. Bul., 15, p. 38.

——— Sheep Poisoning. Rocky Mountain Husbandman, October 27, 1898.

Wildt, E. Kritische Bemerkungen zu eigigen Methoden der Lupinenentitterung. Biedermann's Centbl. Agr. Chem., 9 (1880), pp. 434-436. (Abstract.)

——— Zur Entbitterung der Lupinen und Unschädlichmachung der in denselben auftretenden giftig wirkenden Stoffe durch Oxydationsmittel. Biedermann's Centbl. Agr. Chem., 13 (1884), pp. 675–677. (Abstract.)

Winterstein, E. Zur Kenntniss der Muttersubstanzen des Holzgummis. Ztschr. Physiol. Chem., 17 (1893), pp. 381–390.

Soll man die Lupinen zur Gründungung verwenden oder reif werden lassen? Landw. Wchnbl. Schleswig-Holstein, 43 (1893), pp. 433, 434.

Studio chimico, esperienze, fisiologische ed applicazioni cliniche sulla lupiniana. Gaz. Chim. Ital., 11 (1881), pp. 240–242.

Ueber Entbitterung und Entgiftung der Lupinenkörner. Deut. Landw. Presse, 23 (1896), Nos. 17, 19.

Verdorbene Lupinen als Dünger. Illus. Landw. Ztg., 19 (1899), No. 28, p. 297.

Nouveau cours complet d'agriculture. Sec. d'Agr. Inst., de France, 8 (1809), pp. 78–82.

Bericht über die Thätigkeit der landwifthschaftlichen Versuchstation an der Universität Jena für das Jahr, 1898, p. 24.

